# Acute kidney injury after nephrectomy: a new nomogram to predict postoperative renal function

**DOI:** 10.1186/s12882-020-01839-0

**Published:** 2020-05-14

**Authors:** Lingyu Xu, Chenyu Li, Long Zhao, Bin Zhou, Congjuan Luo, Xiaofei Man, Hong Luan, Lin Che, Yanfei Wang, Yan Xu

**Affiliations:** Department of Nephrology, The Affiliated Hospital of Qingdao University, 16 Jiangsu Road, Qingdao, 266003 China

**Keywords:** Nephrectomy, Renal function, Acute kidney injury, Predictor, Nomogram, Prognosis

## Abstract

**Background:**

We aimed to develop a nomogram based on preprocedural features for early prediction of acute kidney injury (AKI) and to assess the prognosis in patients after radical and partial nephrectomy.

**Methods:**

The study included a development cohort of 1111 patients who were treated between June 2012 and June 2017 and an additional validation cohort of 356 patients who were treated between July 2017 and June 2018. Stepwise regression and logistic regression analyses were used to evaluate the association between predictors and AKI. Incorporating all independent predictors, a nomogram for postoperative AKI was developed and externally validated. Patients were followed up for 5 years to assess renal function, acute kidney disease (AKD), chronic kidney disease (CKD), hospital readmission and mortality were key prognosis we focused on.

**Results:**

After multivariate logistic regression, radical nephrectomy (odds ratio (OR) = 3.57, *p* < 0.001), aspirin (OR = 1.79, *p* = 0.008), systolic blood pressure (OR = 1.41, *p* = 0.004), triglyceride (OR = 1.26, *p* = 0.024), and alkaline phosphatase (OR = 1.75, *p* = 0.034) were independent risk factors for postoperative AKI, while albumin (OR = 0.72, *p* = 0.031) was a protective factor for postoperative AKI. Patients with a higher estimated glomerular filtration rate (eGFR) (60–90 ml/min/1.73 m^2^, OR = 0.41, *p* = 0.004; ≥ 90 ml/min/1.73 m^2^, OR = 0.37, *p* < 0.001) were less prone to AKI than those with a lower eGFR (< 15 ml/min/1.73 m^2^). These predictors were all included in the final nomogram. The area under the receiver operating characteristics curve for the model were 0.77 (*p* < 0.001) in the development cohort and 0.72 (*p* < 0.001) in the validation cohort. The incidence of AKD and CKD were 27.12 and 18.64% in AKI group, which were much higher than those in no AKI group (*p* < 0.001).

**Conclusions:**

The nomogram had excellent predictive ability and might have significant clinical implications for the early detection of AKI in patients undergoing nephrectomy.

## Background

Renal masses are a biologically heterogeneous group of tumors ranging from benign masses to cancers that are commonly encountered in urologic practice; renal cell carcinoma (RCC) is the most lethal urological malignancy, and angiomyolipoma is the most common benign entity [1–3]. The standard treatment is surgical excision, including radical nephrectomy (RN) or partial nephrectomy (PN) and laparotomy or minimally invasive approaches [4, 5]. RN has been the standard surgical therapy for localized renal masses for many decades [6–8], even though this treatment may lead to a rapid decline in renal function and an increased risk of chronic kidney disease (CKD), which causes death in the short or long term [9–14]. In contrast, PN is more effective for preserving renal function [15–17]. In recent years, abdominal computed tomography and magnetic resonance imaging have made detection of smaller renal masses possible, which assists urologists in conducting early clinical interventions before tumor progression and metastasis. Therefore, compared to RN, which involves removal of the whole kidney, PN is emphasized in the management of patients with clinical T1 renal masses that can be cured [10, 13], while RN remains the standard surgery for tumors > 7 cm (T2 or more) [4, 17, 18]. In addition to PN and RN, there has also been an evolution in surgical management, and minimally invasive nephrectomy has gained importance as an alternative treatment to laparotomy.

Although surgery may become the preferred choice for urologists, studies have reported that nephrectomy is associated with a high iatrogenic risk of acute kidney injury (AKI) [11, 19–21]. AKI, a serious medical condition and complication characterized by persistent oliguria and elevated serum creatinine (Scr) level, is associated with high morbidity and mortality, especially after surgery and in the intensive care unit (ICU) [22, 23]. The pathophysiology of postoperative AKI involves various factors, and previous studies have developed several mathematical models to assess the risk of AKI. These proposed models were postprocedural and contained some intraoperative factors, such as anesthesiologists’ score and operative duration. Patients must undergo surgery to have this information obtained [21, 24–26], which is untimely to conduct clinical intervention. From our perspective, there is no conclusion regarding which factor can lead to AKI after nephrectomy, and identifying risk factors may aid in the application of preoperative preventative methods for patients. Therefore, a risk model with quick applicability to assess AKI risk prior to the procedure may be timely and clinically important. This is a pioneering study to assess various predictors and to develop a model for the prediction of postoperative AKI before nephrectomy. And patients were followed up for 5 years to analyze the incidence of acute kidney disease (AKD), CKD, hospital readmission and death.

A nomogram is a visual tool that has been increasingly utilized to visualize an approximate graphical calculation of regression models, which can assist clinicians in predicting patient outcomes and making clinical decisions. Based on logistic regression, we innovatively used a nomogram to explore the relationship between predictors and postoperative AKI. By integrating these clinical factors, a nomogram can provide individualized estimates of the probability of an outcome. Therefore, these algorithms might be used as reliable tools for predicting AKI and guiding decisions regarding nephrectomy.

## Methods

### Study population and data collection

Our hospital is one of the top 100 hospitals in China. Its subordinate urology department, the provincial key discipline, has 7 high level physicians with more than 10 years of surgical experience, and more than 400 cases of nephrectomy are performed each year. The development cohort included patients who underwent nephrectomy between June 2012 and June 2017, while the validation cohort was collected between July 2017 and June 2018. We further study 5-year prognosis of patients after nephrectomy, AKD, CKD, hospital readmission and mortality were key outcomes we focused on. Patients were excluded if they met one of the following characteristics:

1. Patients with radical nephroureterectomy or renal cystic unroofing.
2. Patients with a duplex kidney or solitary kidney.
3. Patients requiring dialysis before nephrectomy.
4. Patients with donor nephrectomies.
5. Patients with missing data elements needed further analysis.

### Covariates

First, we assessed the demographic information of all patients: age at surgery, gender, body mass index (BMI), smoking and alcohol status. Additionally, preoperative data, such as routine blood tests, coagulation markers, blood biochemical examinations, urinalyses, and blood pressure measurements were collected simultaneously. Operative information included the procedure type (RN and PN), approach (laparoscopic, open, or robotic) for nephrectomy. Comorbidities and the usage of drugs, such as angiotensin-converting enzyme inhibitors/angiotensin receptor blockers (ACEIs/ARBs), calcium channel blocker (CCB), β-adrenergic antagonists, nonsteroidal anti-inflammatory drugs (NSAIDs), aspirin, aminoglycoside antibiotics, quinolones, β-lactams, proton pump inhibitor (PPI), and metformin were also collected. Moreover, prognosis (AKD, CKD, hospital readmission and mortality) were followed up as well. The estimated glomerular filtration rate (eGFR) was calculated using the Chronic Kidney Disease Epidemiology Collaboration (CKD-EPI) creatinine formula, a 4-variable equation consisting of age, race, gender, and Scr level [27, 28]. Other details are listed in Table 1.

**Table 1:** Characteristics of 1111 patients at risk of AKI after nephrectomy in development cohort

Variables	Total no. (%)	No AKI no. (%)	AKI no. (%)	t/χ^2^	*P*-value
No.of patients	1111 (100)	875 (78.76)	236 (21.24)	–	–
Age, y, mean (SD)	55.17 (12.59)	54.56 (12.49)	57.47 (12.70)	3.17	**0.002**
Gender					
Female	472 (42.48)	398 (45.49)	74 (31.36)	15.19	**< 0.001**
Male	639 (57.52)	477 (54.51)	162 (68.64)		
BMI, kg/m^2^, mean (SD)	25.12 (3.33)	25.00 (3.33)	25.56 (3.28)	2.28	**0.023**
Procedure					
Partial nephrectomy	360 (32.40)	314 (35.89)	46 (19.49)	22.81	**< 0.001**
Radical nephrectomy	751 (67.60)	561 (64.11)	190 (80.51)		
Approach					
Laparoscopic	821 (73.90)	647 (73.94)	174 (73.73)	0.26	0.877
Open	204 (18.36)	162 (18.51)	42 (17.80)		
Robotic	86 (7.74)	66 (7.54)	20 (8.47)		
Baseline laboratory values					
Mean PLT, × 10^9^/L (SD)	245.45 (76.70)	246.48 (76.89)	241.60 (76.07)	0.87	0.390
Mean FIB, g/L (SD)	3.18 (1.04)	3.18 (1.07)	3.21 (0.96)	0.37	0.712
Mean PCT, ng/ml (SD)	0.24 (0.07)	0.24 (0.07)	0.24 (0.07)	0.55	0.582
Mean MPV, fl (SD)	9.83 (1.05)	9.81 (1.06)	9.88 (1.00)	0.84	0.402
Mean Hb, g/L (SD)	135.90 (19.70)	135.92 (19.28)	135.80 (21.22)	0.08	0.930
Mean MCHC, g/L (SD)	333.72 (12.62)	333.88 (12.60)	333.15 (12.71)	0.78	0.434
Mean MCH, pg (SD)	29.82 (2.36)	29.84 (2.32)	30.05 (2.50)	1.34	0.181
Mean MCV, fL (SD)	89.42 (5.57)	89.23 (5.44)	90.11 (6.02)	2.15	**0.032**
Mean ALT, U/L (SD)	21.33 (15.91)	21.30 (16.23)	21.46 (14.67)	0.14	0.886
Mean AST, U/L (SD)	18.32 (8.78)	18.41 (9.35)	17.80 (6.23)	0.64	0.520
Mean STB, μmol/L (SD)	14.98 (6.57)	14.94 (6.57)	15.10 (6.58)	0.33	0.740
Mean CHOL, mmol/L (SD)	5.02 (1.10)	5.06 (1.11)	4.88 (1.05)	2.20	**0.028**
Mean TG, mmol/L (SD)	1.41 (1.09)	1.38 (1.07)	1.51 (1.16)	1.60	0.111
Mean TP, g/L (SD)	68.77 (6.03)	69.00 (6.01)	67.90 (6.03)	2.51	**0.012**
Mean ALB, g/L (SD)	40.06 (4.62)	40.20 (4.62)	39.51 (4.57)	2.04	**0.041**
Mean ALP, U/L (SD)	75.05 (35.52)	74.12 (34.04)	78.46 (40.44)	1.67	0.096
Mean LDH, U/L (SD)	164.75 (60.47)	163.69 (63.09)	168.67 (49.47)	1.29	0.198
Mean HDL, mmol/L (SD)	1.36 (0.34)	1.38 (0.34)	1.30 (0.32)	3.36	**0.001**
Mean TT%, % (SD)	1.07 (0.10)	1.07 (0.10)	1.07 (0.09)	0.46	0.646
Mean TT, s (SD)	14.66 (2.05)	14.75 (2.12)	14.36 (1.74)	2.60	**0.009**
Mean GLU, mmol/L (SD)	5.58 (1.87)	5.59 (1.95)	5.55 (1.53)	0.24	0.811
Mean HCT, % (SD)	40.48 (5.55)	40.49 (5.43)	40.45 (5.97)	0.10	0.921
Mean UA, μmol/L (SD)	320.69 (90.15)	317.21 (89.77)	333.60 (90.60)	2.47	**0.014**
Mean SBP, mmHg (SD)	130.30 (17.37)	129.64 (17.17)	132.76 (17.93)	2.45	**0.014**
Mean DBP, mmHg (SD)	81.11 (10.97)	80.93 (10.73)	81.80 (11.84)	1.09	0.276
Preoperative eGFR, ml/min/1.73 m^2^					
< 60	127 (11.43)	96 (10.97)	31 (13.14)	36.41	**< 0.001**
≥60	984 (88.57)	779 (89.03)	205 (86.86)		
Comorbidities					
Hypertension	333 (29.97)	242 (27.66)	91 (38.56)	10.53	**0.001**
Diabetes mellitus	131 (11.79)	100 (11.43)	31 (13.14)	0.52	0.471
CHD	85 (7.65)	63 (7.20)	22 (9.32)	1.19	0.276
PU	46 (4.14)	35 (4.00)	11 (4.66)	0.21	0.651
FLD	53 (4.77)	39 (4.46)	14 (5.93)	0.89	0.345
CKD	5 (0.45)	0 (0.00)	5 (2.12)	18.62	**< 0.001**
Cystic kidney disease	135 (12.15)	105 (12.00)	30 (12.71)	0.09	0.766
Renal calculi	126 (11.34)	119 (13.60)	7 (2.97)	20.90	**< 0.001**
Hydronephrosis	107 (9.63)	101 (11.54)	6 (2.54)	17.30	**< 0.001**
Drugs					
ACEIs/ARBs	123 (11.07)	92 (10.51)	31 (13.14)	1.30	0.255
CCB	249 (22.41)	178 (20.34)	71 (30.08)	10.14	**0.001**
β-adrenergic antagonists	469 (42.21)	371 (42.40)	98 (41.53)	0.06	0.809
NSAIDs	263 (23.67)	208 (23.77)	55 (23.31)	0.02	0.881
Aspirin	174 (15.66)	129 (14.74)	45 (19.07)	2.63	0.105
Aminoglycoside antibiotics	53 (4.77)	11 (4.66)	42 (4.80)	0.01	0.929
Quinolones	158 (14.22)	131 (14.97)	27 (11.44)	1.90	0.168
β-Lactams	258 (23.22)	199 (22.74)	59 (25.00)	0.53	0.466
PPI	1086 (97.75)	856 (97.83)	230 (97.46)	0.12	0.733
Metformin	35 (3.15)	26 (2.97)	9 (3.81)	0.43	0.511
Life style					
Alcohol	321 (28.89)	233 (26.63)	88 (37.29)	10.28	**0.001**
Smoking	375 (33.75)	286 (32.69)	89 (37.71)	2.10	0.147
Insurance status					
Yes	979 (88.12)	772 (88.23)	207 (87.71)	0.05	0.828
No	132 (11.88)	103 (11.77)	29 (12.29)		
Length of hospital stay	10.99 (3.80)	11.07 (3.60)	10.69 (4.45)	2.86	**0.004**
Prognosis					
AKD	127 (11.43)	63 (7.20)	64 (27.12)	72.84	**< 0.001**
CKD	96 (8.64)	52 (5.94)	44 (18.64)	37.98	**< 0.001**
Hospital readmission	142 (12.78)	74 (8.46)	68 (28.81)	69.09	**< 0.001**

*Note:* numbers in bold mean they are significance (*p* < 0.05)

Abbreviations: *AKI* Acute kidney injury, *SD* Standard deviation, *BMI* Body mass index, *PLT* Platelet, *FIB* Fibrinogen, *PCT* Platelet crit, *MPV* Mean platelet volume, *Hb* Hemoglobin, *MCHC* Mean corpuscular hemoglobin concentration, *MCH* Mean corpuscular hemoglobin, *MCV* Mean corpuscular volume, *ALT* Alanine transaminase, *AST* Aspartate transaminase, *STB* Total bilirubin, *CHOL* Cholesterol, *TG* Triglyceride, *TP* Total protein, *ALB* Albumin, *ALP* Alkaline phosphatase, *LDH* Lactate dehydrogenase, *HDL* High density lipoprotein, *TT* Thrombin time, *GLU* Glucose, *HCT* Hematocrit, *UA* Uric acid, *SBP* Systolic blood pressure, *DBP* Diastolic blood pressure, *eGFR* Estimated glomerular filtration rate, *CHD* Coronary heart disease, *PU* Peptic ulcer, *FLD* Fatty liver disease, *CKD* Chronic kidney disease, *ACEIs* Angiotensin-converting enzyme inhibitors, *ARBs* Angiotensin receptor blockers, *CCB* Calcium channel blocker, *NSAIDs* Non-steroidal anti-inflammatory drugs, *PPI* Proton pump inhibitor, *AKD* Acute kidney disease

### Outcome definition

The primary endpoint was postoperative AKI, which identified based on latest Kidney Disease: Improving Global Outcomes (KDIGO) Clinical Practice Guideline [29] for AKI: (1) Increase in Scr level by ≥26.5 μmol/L (0.3 mg/dL) within 48 h; (2) Increase in Scr level to ≥1.5 times baseline, which is known or presumed to have occurred within the prior 7 days (Additional file 1). The most recent Scr level before nephrectomy was selected as the baseline Scr. The second endpoint was AKD, CKD, hospital readmission and mortality. AKD was defined as a condition in which AKI stage 1 or greater was present ≥7 days after an AKI initiating event [30], while AKD that persisted beyond 90 days was considered to be CKD [31].

### Statistical analysis

Numerical variables are expressed as the mean ± standard deviation (SD), while descriptive statistics of categorical variables are reported as frequencies and proportions. Continuous and categorical variables were compared by Student’s t-test and the χ^2^-test or Fisher’s exact test, respectively. For further analyses, continuous variables were transformed into categorical variables. Then, we performed stepwise and logistic regression analyses with postoperative AKI as the dependent variable, and the results are presented as odds ratios (ORs) and 95% confidence intervals (CIs). Incorporating all independent predictors, a logistic regression-based nomogram to predict the risk of postoperative AKI was developed and externally validated using the validation cohort. Survival analysis was used to assess prognosis and multiple imputation was used to estimate missing data. All statistical analyses were performed using the Statistical Package SPSS (version 23.0, SPSS Inc., Chicago, IL, USA) and R software (The R Foundation for Statistical Computing, www.R-project.org), with a 2-sided significance level set at *p* < 0.05.

## Results

### Baseline characteristics

**Fig. 1:**
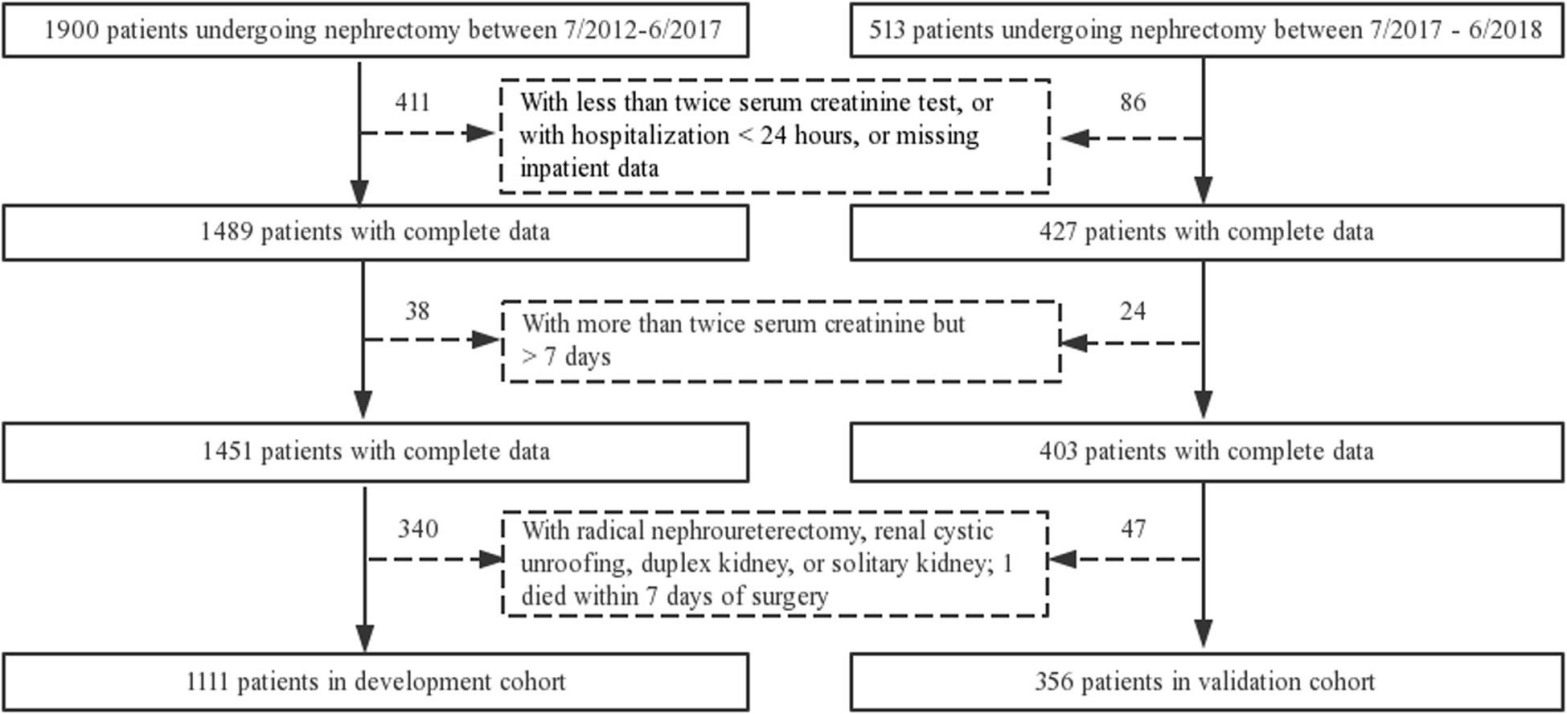
Flow diagram of patients enrollment

Initially, the development cohort included 1900 patients who underwent nephrectomy between June 2012 and June 2017, and the additional validation cohort consisted of 513 patients who were treated between July 2017 and June 2018. After screening based on the exclusion criteria, the remaining 1111 (58.47%) and 356 (69.40%) patients were included in the final analyses (Fig. 1). From 2013 to 2016, 941 patients underwent nephrectomy. Among them, RN (68.23%) was more than two times as frequently as PN (31.77%), and laparoscopic RN was the predominant treatment approach. Because of the introduction of robotic surgical systems, the use of robotic PN (0 to 8.15%) increased year by year, whereas the rates of open PN (1.75 to 1.69%) and laparoscopic PN (26.32 to 26.12%) remained relatively unchanged. (Additional file 2).

Patients underwent RN were more susceptible to postoperative AKI than PN patients (*p* < 0.001). According to baseline laboratory values, RN patients had higher platelet (PLT) counts, fibrinogen (FIB), alkaline phosphatase (ALP), and lactate dehydrogenase (LDH) levels (*p* < 0.01), while patients who underwent PN had higher hemoglobin (Hb), mean corpuscular hemoglobin concentration (MCHC), mean corpuscular hemoglobin (MCH), cholesterol (CHOL), triglyceride (TG), albumin (ALB), and hematocrit (HCT) levels (*p* < 0.05), indicating that RN patients might have worse preoperative physical conditions than patients who underwent PN. Moreover, patients with a preoperative eGFR < 60 ml/min/1.73 m^2^ were more commonly represented in the RN group than in the PN group (13.98% vs. 6.11%, *p* < 0.001), showing worse preoperative renal function among RN patients. (Table 1 and Additional file 3).

### Outcome of renal function

In the development cohort, 236 patients (21.24%) experienced postoperative AKI. AKI patients were more likely than patients with normal postoperative kidney function to have undergone RN rather than PN (80.51% vs. 64.11%, *p* < 0.001), but the surgical approach between the two groups was not statistically significant (*p* > 0.05). The mean preoperative thrombin time (TT), mean corpuscular volume (MCV), CHOL, total protein (TP), ALB, high density lipoprotein (HDL), uric acid (UA), and systolic blood pressure (SBP) significantly differed between the patients with and without AKI (*p* < 0.05), AKI group seemed to have a higher but relatively normal BMI level (*p* = 0.023). Compared to patients without postoperative AKI, a greater proportion of AKI patients had a preoperative eGFR < 60 ml/min/1.73 m^2^ (13.14% vs. 10.97%, *p* < 0.001). Moreover, there were 5 patients with CKD before nephrectomy who all developed AKI after surgery. All of those indicated that a relatively normal preoperative renal function might prevent patients from developing postoperative AKI. We also compared the usage of clinical drugs between different groups. Relative to patients with normal postoperative kidney function, AKI patients were more likely to use CCB (30.08% vs. 20.34%, *p* < 0.05), but it was excluded from the final model by stepwise regression analysis. RN patients were more likely to use aminoglycoside antibiotics, while more proportion of PN patients use aspirin (*p* < 0.05). (Table 1 and Additional file 3).

**Fig. 2:**
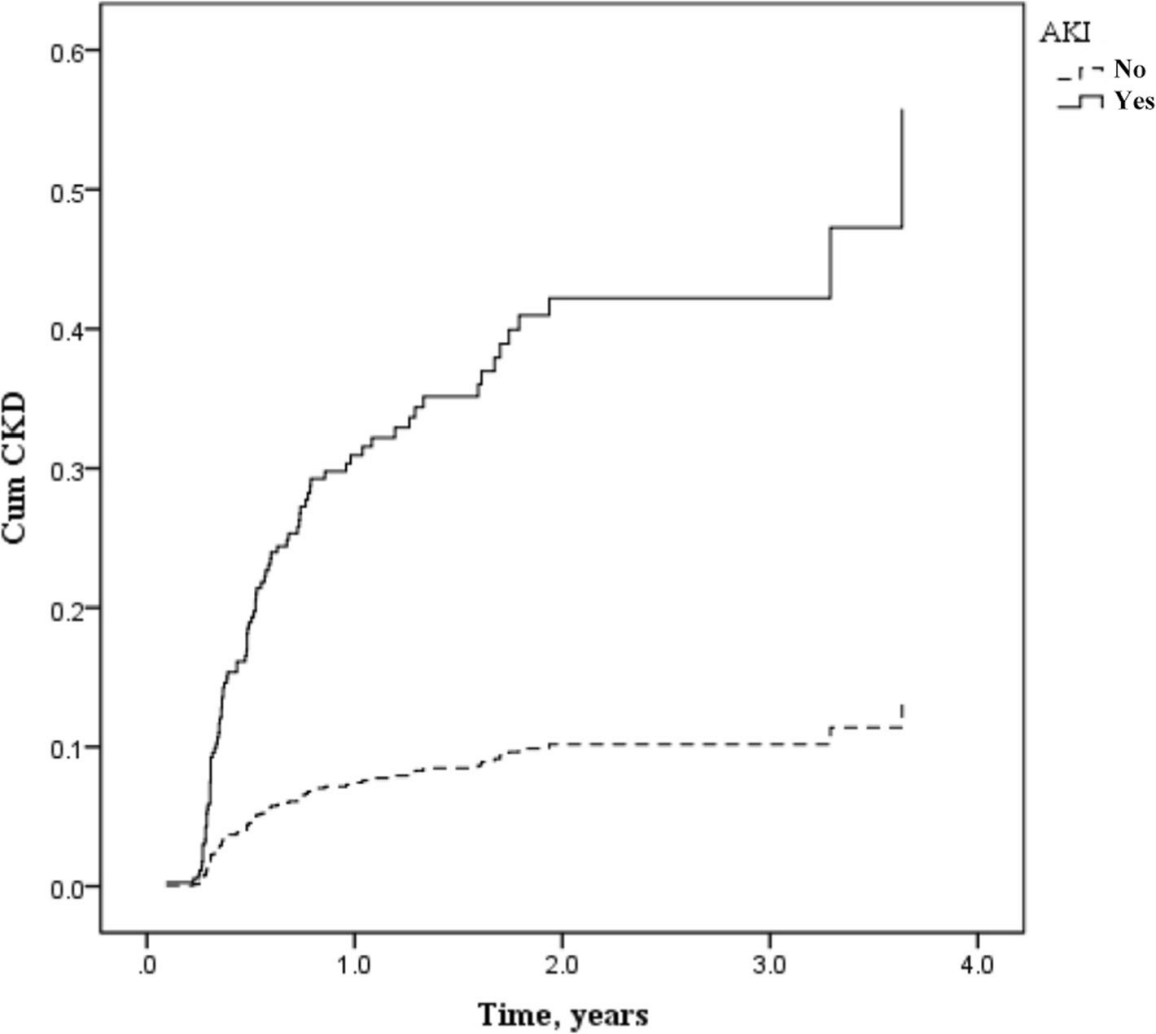
CKD probabilities between AKI and no AKI patients during follow-up period

We further study 5-year prognosis of patients after nephrectomy. There were 64 (27.12%) AKI patients who developed AKD, while only 63 (7.20%) in no AKI group, and the hospital readmission rate of AKI patients was also higher than without AKI (28.81% vs. 8.46%). Moreover, 44 AKI patients developed CKD during follow-up, which was much higher than that in no AKI group (18.64% vs. 5.94%, *p* < 0.05) (Fig. 2). There was only one patient who developed postoperative AKI died during follow-up. All of these results indicated that postoperative AKI might worse prognosis after nephrectomy.

### Multivariable analysis

Multivariable logistic regression analysis showed that male gender (OR = 1.68, 95%CI: 1.20–2.35, *p* = 0.002), RN (OR = 3.57, 95%CI: 2.42–5.26, *p* < 0.001), aspirin (OR = 1.79, 95%CI: 1.17–2.75, *p* = 0.008), high PT% (OR = 2.46, 95%CI: 1.15–5.24, *p* = 0.020), TG (OR = 1.26, 95%CI: 1.03–1.53, *p* = 0.024), ALP (OR = 1.75, 95%CI: 1.04–2.94, *p* = 0.034), and SBP (OR = 1.41, 95%CI: 1.12–1.78, *p* = 0.004) were independent risk factors for postoperative AKI, while ALB (OR = 0.72, 95%CI: 0.54–0.97, *p* = 0.031), TT (OR = 0.65, 95%CI: 0.47–0.89, *p* = 0.007), platelet crit (PCT, OR = 0.47, 95%CI: 0.29–0.78, *p* = 0.003) were protective factors for postoperative AKI. An eGFR of 60–90 ml/min/1.73 m^2^ (OR = 0.41, 95%CI: 0.22–0.76, *p* = 0.004) or ≥ 90 ml/min/1.73 m^2^ (OR = 0.37, 95%CI: 0.26–0.53, *p* < 0.001) effectively prevented postoperative AKI compared with an eGFR < 15 ml/min/1.73 m^2^. More details are shown in Table 2.

**Table 2:** Multivariable logistic regression analysis for predictors of postoperative AKI

Variables	OR (95% CI)	*p*-value
Gender		
Female	1.00	
Male	1.68 (1.20, 2.35)	**0.002**
Procedure		
Partial nephrectomy	1.00	
Radical nephrectomy	3.57 (2.42, 5.26)	**< 0.001**
Baseline laboratory values		
Mean TT, s	0.65 (0.47, 0.89)	**0.007**
Mean PCT, %	0.47 (0.29, 0.78)	**0.003**
Mean PT%, %	2.46 (1.15, 5.24)	**0.020**
Mean ALB, g/L	0.72 (0.54, 0.97)	**0.031**
Mean TG, mmol/L	1.26 (1.03, 1.53)	**0.024**
Mean ALP, U/L	1.75 (1.04, 2.94)	**0.034**
Preoperative eGFR, ml/min/1.73 m^2^		
< 15	1.00	
15–30	2.18 (0.10, 47.67)	0.620
30–45	7.14 (0.80, 63.58)	0.078
45–60	1.53 (0.55, 4.24)	0.416
60–90	0.41 (0.22, 0.76)	**0.004**
≥90	0.37 (0.26, 0.53)	**< 0.001**
SBP, mmHg	1.41 (1.12, 1.78)	**0.004**
Comorbidities		
Renal calculi	0.18 (0.08, 0.44)	**< 0.001**
Hydronephrosis	0.23 (0.09, 0.56)	**0.001**
Drug		
Aspirin	1.79 (1.17, 2.75)	**0.008**

*Note:* numbers in bold mean they are significance (*p* < 0.05)

Abbreviations: *AKI* Acute kidney injury, *OR* Odds ratio, *95% CI* 95% confidence interval, *TT* thrombin time, *PCT* Platelet crit, *PT* Prothrombin time, *ALB* Albumin, *TG* Triglyceride, *ALP* Alkaline phosphatase, *eGFR* Estimated glomerular filtration rate, *SBP* Systolic blood pressure

### Nomogram development

**Fig. 3:**
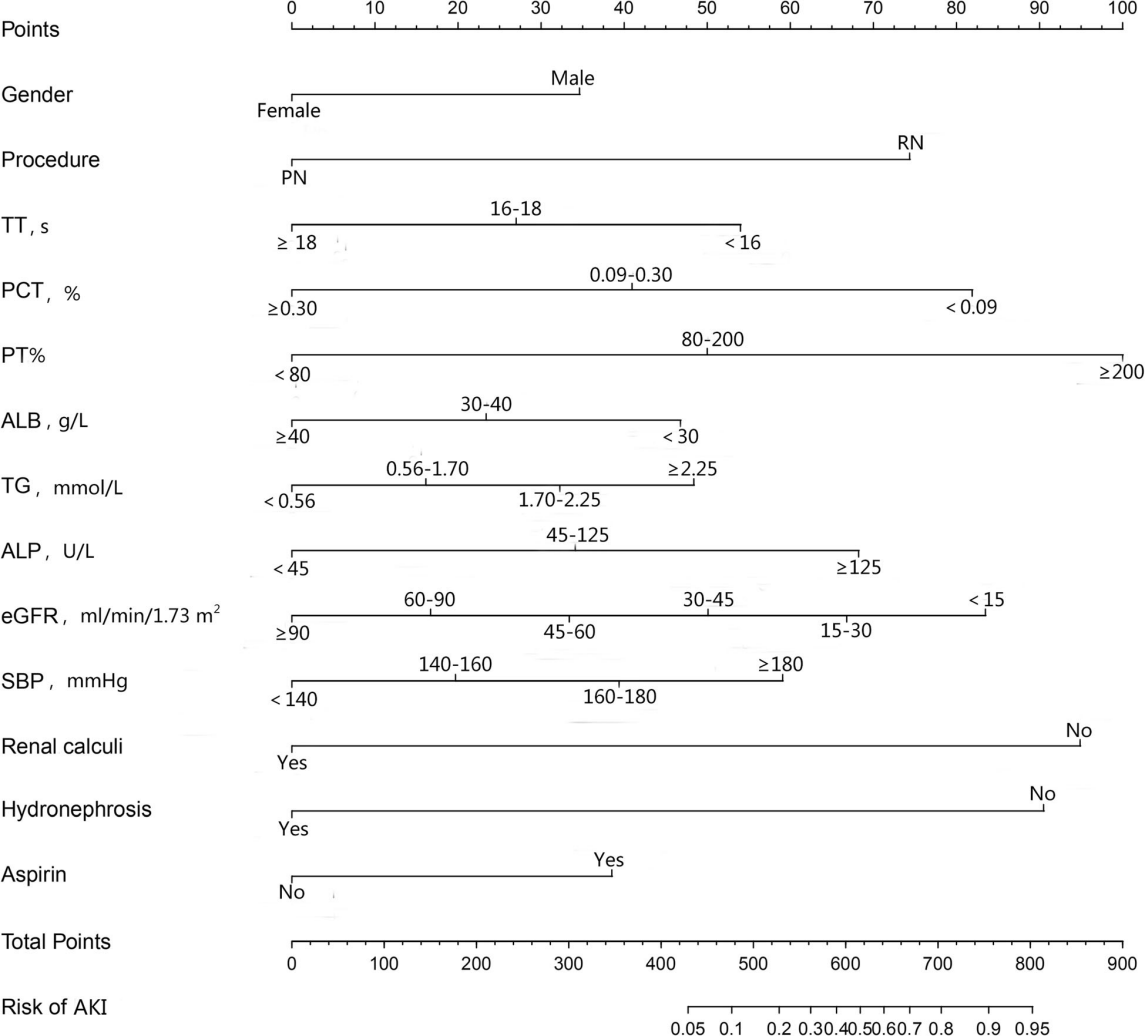
A logistic regression-based nomogram to predict the possibility of AKI after nephrectomy. To obtain the predicted probability of postoperative AKI, locate patient values on each axis. Draw a vertical line upward to the “Points” axis to determine the points of the variable. Sum the points for all variables and locate the sum on the “Total Points” axis. Draw a vertical line down to the “Risk of AKI” axis to find the patient’s possibility of AKI after nephrectomy. The predicted range of developing postoperative AKI was from 5 to 95%

**Fig. 4:**
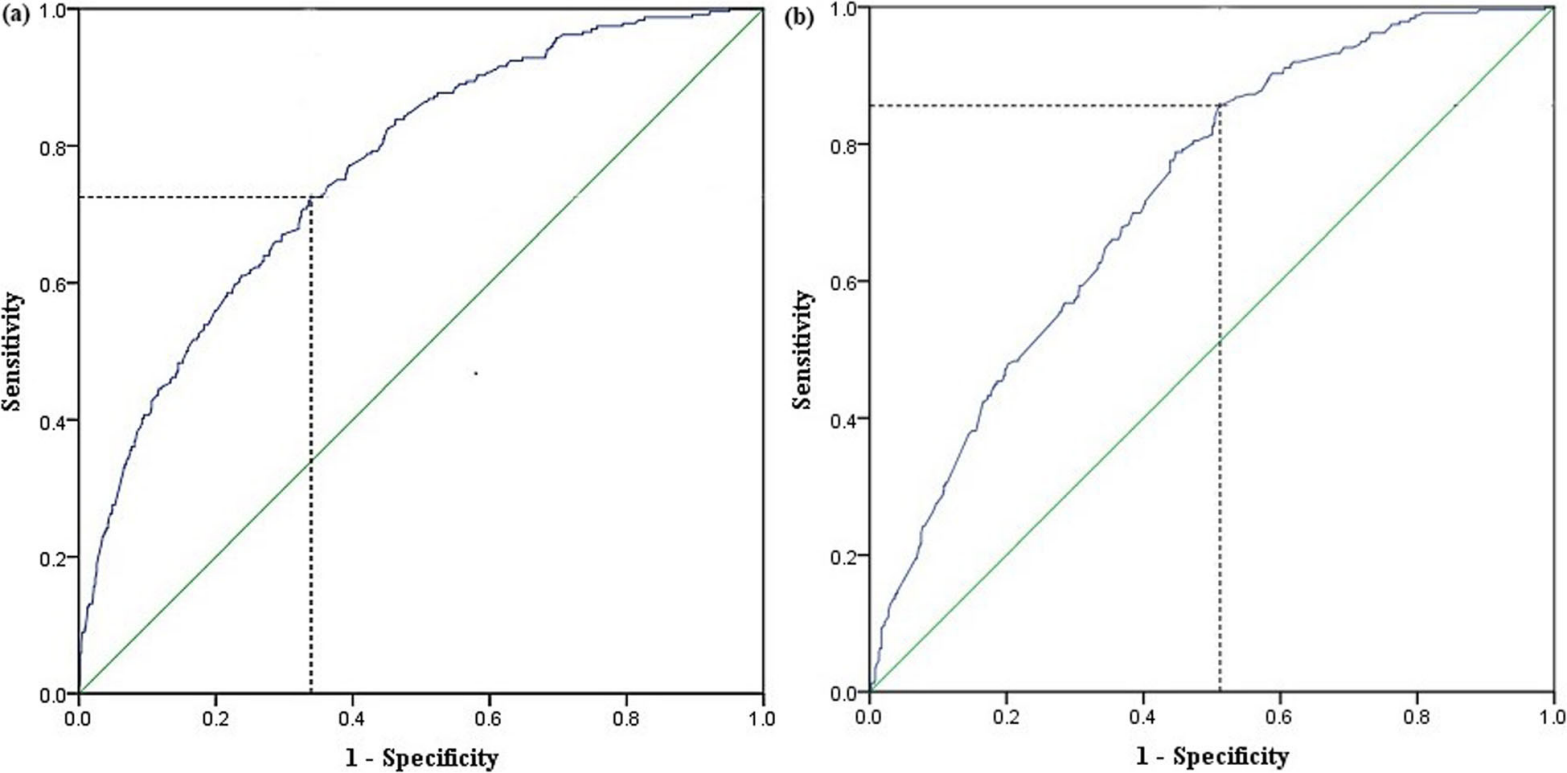
Internal and external validation of the nomogram to predict the possibility of AKI after nephrectomy. The area under the receiver operating characteristic curve (AUC) were 0.77 (95% CI: 0.73–0.80, *p* < 0.001) in development cohort (**a**) and 0.72 (95% CI: 0.69–0.76, *p* < 0.001) in validation cohort (**b**)

A nomogram (Fig. 3) to predict the possibility of postoperative AKI before patients undergoing nephrectomy was developed using the results from multivariate logistic regression. Points were assigned to the thirteen identified factors according to their regression coefficients. The nomogram was internally and externally validated, and the discriminative ability was evaluated using the area under the receiver operating characteristics curve (AUC), which was 0.77 (95% CI: 0.73–0.80, *p* < 0.001) in the development cohort and 0.72 (95% CI: 0.69–0.76, *p* < 0.001) in the external validation cohort. The sensitivity and specificity of the development model were 0.73 and 0.66, and 0.86 and 0.49 in the validation cohort, indicating that the model could accurately predict postoperative AKI using preoperative predictors noted above. (Fig. 4).

## Discussion

During the last 20 years, the incidence of solid renal masses has been increasing, regardless of whether the mass was benign or malignant, the main treatment was nephrectomy. However, postoperative renal dysfunction is still a constant concern faced by healthcare providers. Firstly, we focused on the short term outcome of AKI after nephrectomy by establishing a predictive model to explore which factors could promote its occurrence and development. Then, long term outcomes (AKD, CKD and hospital readmission) were also collected to assess prognosis of patients underwent nephrectomy. The incidence of postoperative AKI in our study was 21.24%, which was relatively high compared with other studies [17, 21, 32]. And the incidence of AKD, CKD and hospital readmission in AKI patients were much higher than people without AKI after nephrectomy, indicated that postoperative AKI could accelerate the deterioration of renal function, which did worse the prognosis of nephrectomy. Compared to PN, RN patients had a larger proportion of preoperative eGFR < 60 ml/min/1.73 m^2^ (13.98% vs. 6.11%, *p* < 0.001), and RN patients seemed to be older than PN group. After logistic regression analysis, RN was associated with a more than 3-fold increase in the odds of postoperative AKI than PN and was identified as an independent risk factor for AKI. The result was consistent with studies in which RN was associated with a greater risk of CKD, cardiovascular events, and even death, while better preservation of renal function after PN had been recognized to improve prognosis after nephrectomy [21, 33]. Therefore, urologists might be more inclined to choose PN for patients with smaller renal masses. The over use of RN should not be recommended for patients who have a low risk of postoperative AKI.

Many studies have been conducted to explore which factors can precisely predict postoperative AKI [21, 24], but the results are inconsistent. In this study, we created a prognostic model, including comprehensively preoperative predictors, to identify the risk of AKI after nephrectomy, which could assist urologists in taking timely clinical intervention before surgery. Among predictors identified by regression analyses, we found that a high SBP and male were risk factors for postoperative AKI, which was consistent with the findings of a previous study [34, 35], reminding urologists to control blood pressure at a safe level, especially for hypertensive patients. Another significant independent predictive factor for outcome was the preoperative eGFR. An eGFR ≥60 ml/min/1.73 m^2^ before surgery could decrease the risk of postoperative AKI. Therefore, it is necessary for clinicians to evaluate patients’ renal function before surgery. Preoperative preparation of all patients should include optimization of renal function, which might become an important measure to improve prognosis.

Renal function after nephrectomy is multifactorial and depends on presurgical factors. Therefore, our study comprehensively analyzed the relationship between preoperative laboratory values and postoperative AKI. After regression analysis, low TT, PCT, and ALB and high TG, PT%, and ALP could slightly increase the risk of postoperative AKI but not as significantly as the eGFR. Hypoalbuminemia was a significant risk factor for AKI and death following AKI. The renal-protective potential of ALB includes mitigation of the nephrotoxicity of medications, protection against loss of the glycocalyx, and maintenance of glomerular filtration [36]. ALP, an endogenous enzyme, exerts detoxifying effects through the dephosphorylation of endotoxins. Study showed that ALP treatment improved renal function in sepsis-induced AKI [37], indicating a protective effect after nephrectomy. Unlike data reported in a sepsis-induced AKI study, we found that the ALP level was increased in the AKI group. This finding might be due to the activation of the host defense mechanism, which promotes ALP activity at an early stage. Hypertriglyceridemia, the major type of dyslipidemia, was an independent risk factor for AKI in the early phase of acute pancreatitis [38], which can be improved by statins [39]. In our study, TG was related to postoperative AKI, which should attract attention as well.

Our study comprehensively analyzed the relationship between clinical drugs and postoperative AKI. Between the AKI and AKI free groups, there were no differences in the use of ACEIs/ARBs, β-adrenergic antagonists, PPI, NSAIDs, aminoglycoside antibiotics and other antibiotics noted above (*p* > 0.05), thus those drugs were excluded from the final model. AKI patients seemed to be more likely to use CCB (30.08% vs. 20.34%, *p* < 0.05), but it was excluded by stepwise regression. We further study the use of drugs in RN patients (Additional file 4), the results showed patients with preoperative eGFR < 60 ml/min/1.73 m^2^ were more likely to use ACEIs/ARBs, CCB, β-adrenergic antagonists (*p* < 0.05), but all of them were excluded by regression analysis (Additional file 5). It should be noted that aspirin was found harm kidney function. Aspirin, an essential medication to prevent cardiovascular disease, is known for its anti-inflammatory and antiplatelet effects. Preoperative low-dose aspirin administration was protective against AKI after cardiac surgery [40], but Sun et al reported that perioperative use of aspirin did not reduce the risk of postoperative AKI and also increased the risk of major bleeding [41]. We suggesting that there is a potential relationship between the dosage of aspirin and AKI, which requires further experiments to investigate.

Finally, based on traditional logistic regression analysis, the nomogram provides a visual method for readers to understand how the predictors impact the outcome. Moreover, synthetically analyzing all the included predictors, we could calculate the probability of the outcome exactly for every subject, which made the results more individualized. In our study, the AUC of the nomogram was 0.77 (*p* < 0.001) in the development cohort and 0.72 (*p* < 0.001) in the validation cohort, indicating that the individual probability of postoperative AKI could be predicted accurately.

The diagnosis of AKI, AKD or CKD rely on a change of Scr concentration or urine output over a defined time interval, because that preoperatively dialysis would affect Scr level [42], which could further influence the diagnosis of our outcomes, we excluded patients requiring dialysis before nephrectomy in our study. After analysis, we found that AKI patients had higher risk of developing CKD. Based on that dialysis was considered to be a main measure for patients with end-stage renal disease [43, 44], we guessed that postoperative AKI would increase the risk of dialysis requirement in the future.

Our study has several limitations. First, this study was conducted at a single institution, and multicenter studies should be conducted in the future. Second, because of the difficulty in data collection, we could not analyze the association between other intraoperative factors and postoperative AKI, such as anesthesiologists’ score, intraoperative hydration status and urine output. However, our study aimed to develop a nomogram based on preprocedural features for early prediction of AKI, which could assist urologists in conducting early clinical interventions before nephrectomy. In addition, due to the lack of drug information, we did not further analyze the relationship between the dosage or duration of drug treatment and outcome. Therefore, more high-quality studies need to be conducted in the future. Third, in our hospital, routine urethral catheterization was conducted within 3 days after nephrectomy, the urine output was accurate during this period. After that, the urine output was recorded by family members of patients, which was not as accurate as the first 3 days. For these reasons, we did not analyze urine output, which were not as objective as blood biomarkers. Fourth, during follow-up, it is very complicated to diagnose and distinguish between AKD without previous AKI and a new AKI episode. Since the definition of initial AKI recovery is lack of consensus and real-time Scr is not easy to get from non-ICU ward.

## Conclusion

In conclusion, we successfully developed and validated a preoperative predictive model, a new nomogram, for AKI after nephrectomy. This predictive model will be helpful in clinical decision making regarding treatment options or follow-up strategies.

## Supplementary information

**Additional file 1.** KDIGO definition and classification of AKI.

**Additional file 2 **Treatment patterns of nephrectomy from 2013 to 2016. *Abbreviations:* LPN, laparoscopicpartial nephrectomy; OPN, open partial nephrectomy; RPN, robotic partial nephrectomy; LRN, laparoscopicradical nephrectomy; ORN, open radical, nephrectomy; RRN, roboticradical nephrectomy.

**Additional file 3.** A total of 1111 patients undergoing partial and radical nephrectomy in development cohort.

**Additional file 4.** Characteristics of 751 patients after radical nephrectomy.

**Additional file 5.** Multivariable logistic regression analysis for predictors of postoperative AKI following radical nephrectomy.

## Data Availability

The datasets used and/or analysed during the current study available from the corresponding author on reasonable request.

## Abbreviations

ACEIs: Angiotensin-converting enzyme inhibitors/angiotensin receptor blockers
AKD: Acute kidney disease
AKI: Acute kidney injury
ALB: Albumin
ALP: Alkaline phosphatase
ALT: Alanine transaminase
ARBs: Angiotensin receptor blockers
AST: Aspartate transaminase
AUC: The area under the receiver operating characteristics curve
BMI: Body mass index
CCB: Calcium channel blocker
CHD: Coronary heart disease
CHOL: Cholesterol
CI: Confidence interval
CKD: Chronic kidney disease
CKD-EPI: The Chronic Kidney Disease Epidemiology Collaboration
DBP: Diastolic blood pressure
DM: Diabetes mellitus
eGFR: estimated glomerular filtration rate
FIB: Fibrinogen
FLD: Fatty liver disease
GLU: Glucose
Hb: Hemoglobin
HCT: Hematocrit
HDL: High density lipoprotein
ICU: Intensive care unit
KDIGO: Kidney Disease Improving Global Outcomes
LDH: Lactate dehydrogenase
MCH: Mean corpuscular hemoglobin
MCHC: Mean corpuscular hemoglobin concentration
MCV: Mean corpuscular volume
MPV: Mean platelet volume
NSAIDs: Non-steroidal anti-inflammatory drugs
OR: Odds ratio
PCT: Platelet crit
PLT: Platelet
PN: Partial nephrectomy
PPI: Proton pump inhibitor
PU: Peptic ulcer
RCC: Renal cell carcinoma
RN: Radical nephrectomy
SBP: Systolic blood pressure
Scr: Serum creatinine
SD: Standard deviation
STB: Total bilirubin
TG: Triglyceride
TP: Total protein
TT: Thrombin time
UA: Uric acid
