# Supplementary material for: Acute kidney injury after nephrectomy: a new nomogram to predict postoperative renal function
Source: BMC Nephrol. 2020 May 14;21:181. doi: 10.1186/s12882-020-01839-0 (PMC7227356; doi:10.1186/s12882-020-01839-0)
Supplement: Supplementary file 1 — Additional file 1. KDIGO definition and classification of AKI. [file 12882_2020_1839_MOESM1_ESM.pdf]

Additional file 1 KDIGO definition and classification of AKI

Diagnostic criteria for AKI:

AKI is defined as any of the following:

- Increase in Scr by ≥ 0.3 mg/dl (≥ 26.5 μmol/L) within 48 hours; or
- Increase in Scr to ≥ 1.5 times baseline, which is known or presumed to have occurred within the prior 7 days; or
- Urine volume < 0.5 ml/kg/h for 6 hours.

AKI staging system:

AKI stage	Scr	Urine output
1	1.5 – 1.9 times baseline<br>or<br>≥ 0.3 mg/dl (≥ 26.5 μmol/L) increase	< 0.5 ml/kg/h for 6 – 12 hours
2	2.0 – 2.9 times baseline	< 0.5 ml/kg/h for ≥ 12 hours
3	≥ 3.0 times baseline<br>or<br>Increase in Scr to ≥ 4.0 mg/dl (≥ 353.6 μmol/L)<br>or<br>Initiation of renal replacement therapy<br>or<br>In patients < 18 years, decrease in eGFR to < 35 ml/min per 1.73 m^2	< 0.3 ml/kg/h for ≥ 24 hours<br>or<br>Anuria for ≥ 12 hours

*Abbreviations:*KDIGO, the 2012 Kidney Disease: Improving Global Outcomes; AKI, acute kidney injury; Scr, serum creatinine.
