# Supplementary material for: Acute kidney injury after nephrectomy: a new nomogram to predict postoperative renal function
Source: BMC Nephrol. 2020 May 14;21:181. doi: 10.1186/s12882-020-01839-0 (PMC7227356; doi:10.1186/s12882-020-01839-0)
Supplement: Supplementary file 2 — Additional file 2 Treatment patterns of nephrectomy from 2013 to 2016. Abbreviations: LPN, laparoscopicpartial nephrectomy; OPN, open partial nephrectomy; RPN, robotic partial nephrectomy; LRN, laparoscopicradical nephrectomy; ORN, open radical, nephrectomy; RRN, roboticradical nephrectomy. [file 12882_2020_1839_MOESM2_ESM.pdf]

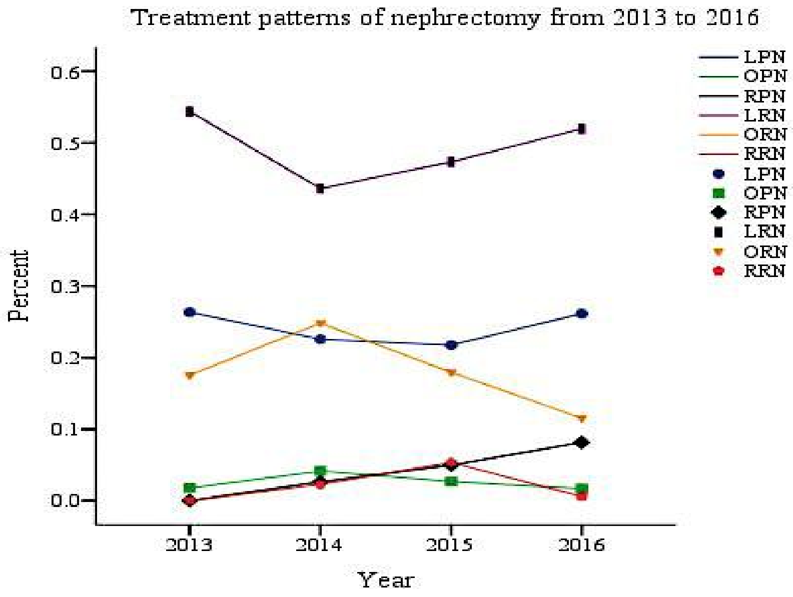

Additional file 2 Treatment patterns of nephrectomy from 2013 to 2016.

Abbreviations: LPN, laparoscopicpartial nephrectomy; OPN, open partial nephrectomy; RPN, robotic partial nephrectomy; LRN, laparoscopicradical nephrectomy; ORN, open radical nephrectomy; RRN, roboticradical nephrectomy.
